# Double methylation of tRNA-U54 to 2′-O-methylthymidine (Tm) synergistically decreases immune response by Toll-like receptor 7

**DOI:** 10.1093/nar/gky644

**Published:** 2018-08-08

**Authors:** Patrick Keller, Isabel Freund, Virginie Marchand, Guillaume Bec, Raven Huang, Yuri Motorin, Tatjana Eigenbrod, Alexander Dalpke, Mark Helm

**Affiliations:** 1 Institute of Pharmacy and Biochemistry, Johannes Gutenberg-University of Mainz, Staudingerweg 5, D-55128 Mainz, Germany; 2 Department of Infectious Diseases, Medical Microbiology and Hygiene, Heidelberg University Hospital, 69120 Heidelberg, Germany; 3 Next Generation Sequencing Platform, UMS2008 Ingénierie Biologie Santé en Lorraine (IBSLor), BioPôle de l’Université de Lorraine Campus Biologie-Santé, 9, avenue de la Forêt de Haye, CS 50184, 54505 Vandoeuvre-les-Nancy, France; 4 Biophysics and Structural Biology Team, Unité Architecture et réactivité de l’ARN (UPR9002), Institut de Biologie Moléculaire et Cellulaire du CNRS, Université de Strasbourg, 15, rue René Descartes, F67084, Strasbourg cedex, France; 5 Department of Biochemistry, Center for Biophysics & Computational Biology, University of Illinois at Urbana-Champaign, 411 Roger Adams Lab., 600 S. Mathews Ave. Urbana, IL 61801, USA; 6 Laboratoire Ingénierie Moléculaire et Physiopathologie Articulaire (IMoPA) UMR7365 CNRS-UL, BioPôle de l’Université de Lorraine Campus Biologie-Santé, 9, avenue de la Forêt de Haye, CS 50184, 54505 Vandoeuvre-les-Nancy, France

## Abstract

Sensing of nucleic acids for molecular discrimination between self and non-self is a challenging task for the innate immune system. RNA acts as a potent stimulus for pattern recognition receptors including in particular human Toll-like receptor 7 (TLR7). Certain RNA modifications limit potentially harmful self-recognition of endogenous RNA. Previous studies had identified the 2′-O-methylation of guanosine 18 (Gm18) within tRNAs as an antagonist of TLR7 leading to an impaired immune response. However, human tRNA^Lys^_3_ was non-stimulatory despite lacking Gm18. To identify the underlying molecular principle, interferon responses of human peripheral blood mononuclear cells to differentially modified tRNA^Lys^_3_ were determined. The investigation of synthetic modivariants allowed attributing a significant part of the immunosilencing effect to the 2′-O-methylthymidine (m^5^Um) modification at position 54. The effect was contingent upon the synergistic presence of both methyl groups at positions C5 and 2’O, as shown by the fact that neither Um54 nor m^5^U54 produced any effect alone. Testing permutations of the nucleobase at ribose-methylated position 54 suggested that the extent of silencing and antagonism of the TLR7 response was governed by hydrogen patterns and lipophilic interactions of the nucleobase. The results identify a new immune-modulatory endogenous RNA modification that limits TLR7 activation by RNA.

## INTRODUCTION

A limited set of germline-encoded pattern recognition receptors is capable of detecting the broad variety of invading pathogens due to the recognition of highly conserved pathogen-associated molecular patterns (PAMPs). Besides cell wall components of bacteria, nucleic acids serve as an important PAMP of viruses as well as of bacteria to activate the innate immune system within minutes. DNA and RNA can either be recognized by endosomal Toll-like receptors (TLR) or cytosolic receptors including retinoic acid-inducible gene I (RIG-I), melanoma differentiation-associated gene 5 (MDA5) and activation of the cGAS –STING axis. Endosomal TLRs like TLR3 and TLR7 are known to sense double-stranded RNA (dsRNA) and single-stranded RNA (ssRNA), respectively (1–4). Upon stimulation of TLR3 or TLR8 in myeloid dendritic (mDCs) cells and macrophages or TLR7 in plasmacytoid dendritic cells (pDCs) type-I interferons are secreted. Several groups consistently reported that, in isolated peripheral blood mononuclear cells (PBMCs), the release of interferon-α (IFN-α) upon stimulation with RNA is attributed to pDCs only (1,5–9).

Previous studies described activation of TLR7 by tRNAs although those RNAs are intrinsically folded and therefore show double-stranded structures. For example, the D-loop and T-loop of canonical tRNAs form very stable tertiary interactions which are reinforced by post-transcriptional modification of key nucleotides (10). Interestingly, one such modification was found to silence the TLR7-mediated response of pDCs toward tRNA of various origins. The highly conserved 2′-O-methylation of guanosine at position 18 is present in ‘self’ as well as ‘non-self’ tRNA isoacceptors (8,11). Given that the residues G18-G19 are buried deep inside the tRNA structure where they are structurally interacting with Ψ55 –C56 (10), TLR7 seems to be able to access residues that are not *a priori* single-stranded. Yet, it is still unclear if recognition of Gm18 by TLR7 is based on an intact tRNA which would imply tRNA unfolding and presumably base-flipping or Gm18 might exhibit its antagonism only after degradation.

Recent crystal structures of the ssRNA sensors TLR7 and TLR8 could only demonstrate the binding of a single nucleotide to a binding site 1 (first) and a tri- or dinucleotide at binding site 2 (second) (12,13). So far, no structure of full-length RNA bound to TLR7 is available, however, triggering TLR7 seems to require a minimum RNA length of ∼20 nt. This piece of biochemical evidence suggests that both binding sites might communicate *via* the continuous chain of a bound RNA (14–16) in turn might trigger TLR7 dimerization. However, because of the degradation of RNAs during the crystallization process, the role of chain connectivity between nucleotides addressing both sites remains unresolved. Of note, inhibition of TRL7 signaling could be achieved with a 2′-O-methylated oligoribonucleotide (17,18) at a minimal length of 9 nt. In addition, small activating molecules such as R848 demonstrate a preference for the first site, illustrating that TLR7 activation can proceed upon binding to site 1 alone (14), which involves an altered signaling cascade (19). The activity of R848 also underscores the importance of hydrophobic interactions within TLR7 (20) and highlights the limit of our understanding with respect to structure–function relationships for activation and inhibition of TLR7 by small molecules, but even more, for RNAs.

The identification of Gm18 as an immunosuppressive modification within tRNAs was initially stimulated by work from Kariko and Weissman (21), who showed that incorporation of certain RNA modifications in *in vitro* transcribed RNA reduced immune-stimulation. Yet, those approaches were done in artificial RNA and considered neither frequency nor position of endogenous, native RNA modifications (22,23). In previous publications, we investigated tRNA as activator of TLR7 for several reasons, namely (i) they comprise a substantial fraction (8–10%) of cellular total RNA, (ii) unlike ribosomal RNA, tRNAs are not tightly bound by proteins, and therefore could be easily accessible for TLR recognition and (iii) bacterial and mammalian tRNAs are extremely similar in both sequence and structure. Of note, tRNAs synthesized by *in vitro* transcription strongly activated TLR7 regardless of the sequence's evolutionary origin, be it prokaryotic or eukaryotic. While isolated eukaryotic tRNAs showed limited activation of TLR7, most isolated bacterial tRNA species were stimulatory (8). This demonstrated a post-transcriptional modification dependent ‘self’ and ‘non-self’ discrimination by TLR7. The non-stimulatory bacterial tRNA^Tyr^ was found to contain Gm18 which was identified as an antagonist of TLR7 and therefore discussed as an immune evasion mechanism (8,17,18,24). Moreover, this work for the first time identified a native inhibitory modification within its natural context. This effect of Gm18 on TLR7 was simultaneously described by the Bauer group (11).

**Figure 1.**
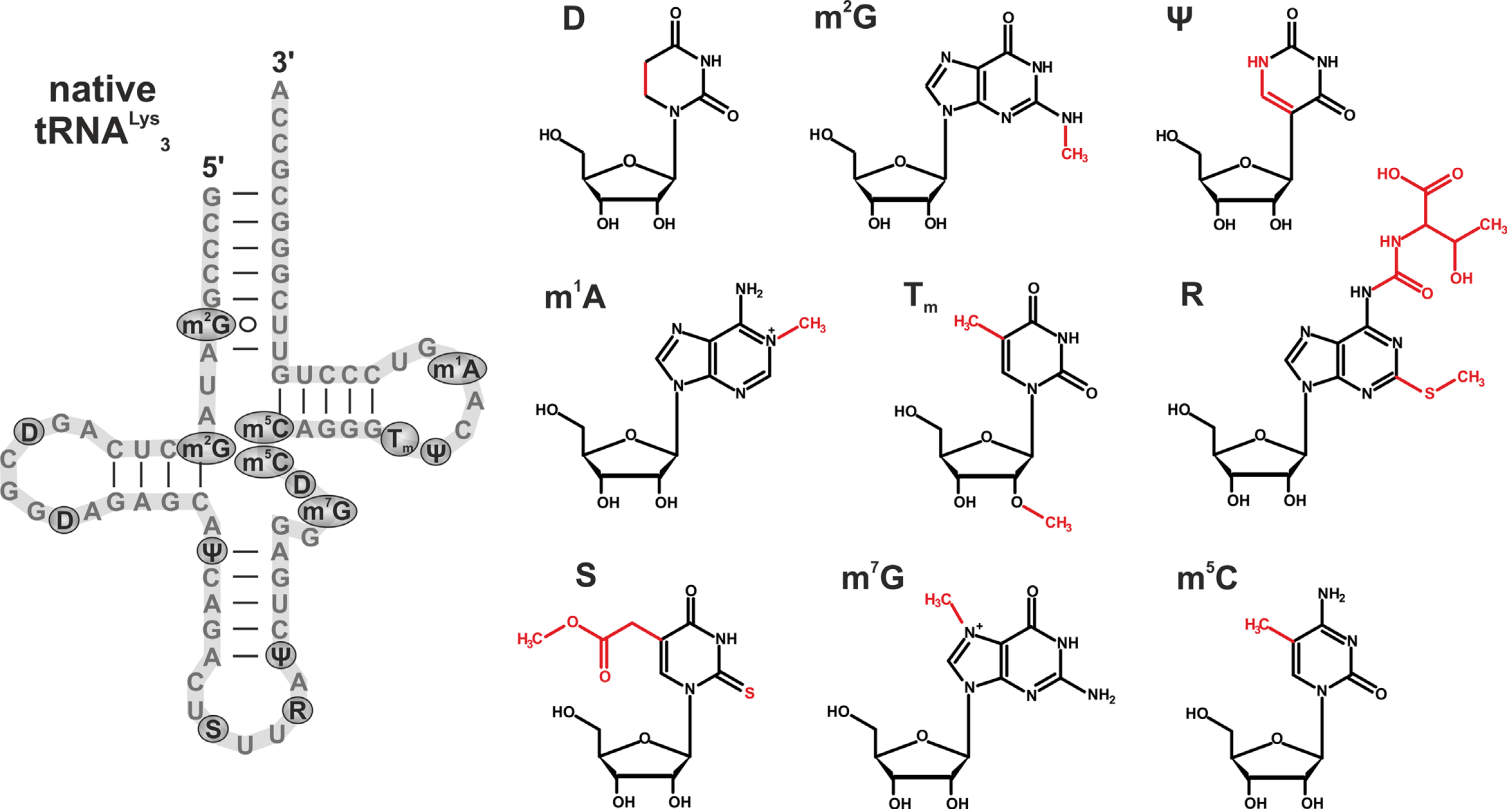
RNA modifications in mammalian tRNA^Lys^_3_. Left part: native tRNA^Lys^_3_ and its corresponding modifications (illustrated as gray circles). Right part: all mentioned modifications and their structural formulas. D = Dihydrouridine, m^2^G = N^2^-methylguanosine, Ψ = Pseudouridine, m^1^A = 1-methyladenosine, Tm = m^5^Um = 2′-O-methylthymidine/5,2′-O-dimethyluridine, R = t^6^A = 2-methylthio-N^6^-threonylcarbamoyladenosine, S = mcm^5^s^2^U = 5-methoxycarbonylmethyl-2-thiouridine, m^7^G = 7-methylguanosine, m^5^C = 5-methylcytidine (according to modomics RNA modification database (40)).

In the course of this project, we observed further non-stimulatory eukaryotic tRNAs, in particular, mammalian tRNA^Lys^_3_, which is depicted along with its post-transcriptional modifications in Figure 1. This tRNA, which holds a special interest in the HIV field (8,22,25–27), appeared to avoid immunostimulation by a novel modification-based principle, given that it does not contain Gm.

To identify the immunosilencing modification pattern, we used a Colicin D-based molecular surgery approach. Colicin D is toxic to infected *Escherichia coli* cells and acts as a so-called tRNase which cleaves the four isoaccepting tRNAs^Arg^*in vivo*, causing an arrest of protein synthesis (28). Here, we adapted its use *in vitro* and found that Colicin D also cleaved mammalian tRNA^Lys^_3_ at a precise position in the anticodon loop. This was exploited to synthesize tRNA^Lys^_3_ modivariants (23). Based on the latter, we could identify that 2′-O-methylthymidine (Tm) is, largely, responsible for the decline of immune response observed in tRNA^Lys^_3_-treated PBMCs.

## MATERIALS AND METHODS

### Cleavage of unmodified/native tRNA^Lys^_3_ by Colicin D

In a final volume of 20 μl, 200 pmoles of unmodified or native RNA were incubated in a solution of Buffer A (final: 5 mM Tris·HCl pH 7.8, 5 mM MgCl_2_, 25 mM KCl) or Buffer C (final: 5 mM HEPES·KOH pH 7.8, 0.5 mM DTT) and 0.8 μg Colicin D (prepared according to (28)) for 1 h at 37°C. The resulting RNA fragment mixture was used without further purification for the hybridization step.

### Hybridization of DNA oligonucleotide to RNA

Hybridization buffer (final: 150 mM KCl, 75 mM HEPES pH 7.0) and an equimolar amount of complementary DNA oligonucleotide (listed below) were added to the fragment mixture. The solution was then incubated for 20 min at room temperature and after that, the DNA/RNA hybrid, as well as the remaining unhybridized RNA fragment were separated via 10% native polyacrylamide gel electrophoresis (PAGE). Bands containing hybrid and fragment were cut out of the gel and agitated in 0.3 M NaOAc overnight at room temperature. In the next step, the RNA was precipitated after addition of two volumes absolute EtOH and glycogen. Followed by resuspension in RNase-free water.

### DNA oligonucleotide used for hybridization

**Table:** 

Name	Description	Sequence (5′-3′)	supplier
MH770	DNA oligo for hybridization to 3′ fragment	TGGCGCCCGAACAGGGACTTGAACCCTGGACCCT	IBA

### DNase treatment and purification of RNA fragments

To obtain the purified, dephosphorylated native fragment, the DNA/RNA hybrid solution was incubated with 0.1 U/μl FastAP (Thermo Scientific) and 1.25 U/μl DNaseI (Thermo Scientific) for 1 h at 37°C. In the next step, the RNA was isolated *via* phenol-chloroform extraction and EtOH precipitation with 0.3 M NaOAc. The concentration of the received fragment in RNase-free water was then checked by UV absorbance.

### Phosphorylation/dephosphorylation of RNA

To remove remaining 2′3′-cyclic phosphates and to get ligation-competent RNA strands (5′ phosphate necessary), 200 pmoles of the purified native fragments or unmodified commercial RNAs were treated with KL-Buffer (final: 10 mM Tris·HCl pH 7.4, 2 mM MgCl_2_), 5 mM DTT, 5 mM adenosine triphosphate (ATP) and 0.75 U/μl T4-Polynucleotide kinase (Thermo Scientific) for 1 h at 37°C. The reaction mixture was used directly for the subsequent splint ligation.

### Splint ligations using tRNA fragments

Unmodified RNAs (see below) were ligated to the corresponding native fragment (Colicin D treatment) or the unmodified 5′ tRNA fragment (Tm modivariant synthesis) to receive a complete tRNA^Lys^_3_ modivariant. For this purpose, prephosphorylated RNA strands were mixed with a 2× molar excess (Colicin D treatment) or an equimolar amount (Tm modivariant synthesis) of the remaining tRNA parts and an equimolar amount of DNA splint in KL-Buffer. After that, the resulting solution was heated for 4 min to 75°C and then slowly cooled down to room temperature for 15 min. In the next step, 1.5 U/μl T4 DNA-Ligase (Thermo Scientific) and 70 ng/μl RNA Ligase 2 (e.g. Thermo Scientific) were added and the mixture was incubated for 24 h at room temperature. At last, the DNA splint was digested (2.5 U/μl DNaseI, 2.5 h at 37°C) and the ligation product was purified using 10% denaturing PAGE (10% acrylamide, 50% urea). The concentration was again checked by UV absorbance.

### Nucleic acids used for splint ligation

**Table:** 

Name	Description	Sequence (5′-3′)	supplier
MH810	tRNA^Lys3^ DNA splint (5′ biotin-, 3′ fluorescein-labeled)	AAATGGCGCCCGAACAGGGACTTGAACCCTGGACCCTCAGATTAAAAGTCTGATGCTCTACCGACTGAGCTATCCGGGC	IBA
MH823	Unmodified 5′ fragment of tRNA^Lys3^ (RNA#2)	GCCCGGAUAGCUCAGUCGGUAGAGCAUCAGACUUUUAA	biomers
MH830	Unmodified 3′ fragment of tRNA^Lys3^	UCUGAGGGUCCAGGGUUCAAGUCCCUGUUCGGGCGCCA	biomers

### Synthesis of 2′-O-methylthymidine bisphosphate (pTmp)

The 5′-3′ bisphosphorylation of the Tm nucleoside was performed as previously described (29). In short, the nucleoside (2 mmoles) was added to a cooled solution of pyrophosphorylchloride (20 mmoles) under an argon atmosphere. Then, the solution was stirred for 5 h at −15°C and after hydrolysis, the evaporated crude product was purified *via* thin layer chromatography (mobile phase: IPA:conc. NH_3_·H_2_O 1:1). In the next step, the bisphosphate was eluted in 0.5 M NH_4_OAc and used as a minimal substrate (30) for mv synthesis. At last, the nucleotide-concentration was determined by UV absorbance. Assignment of product-spots was achieved according to literature (31).

To assure the production of the bisphosphate, an aliquot of pTmp (50 pmoles) was treated with 0.3 U Nuclease P1 (Sigma Aldrich) in 20 μl reaction solution (22.5 mM NH_4_OAc pH 5.0, 20 μM ZnCl_2_) for 30 min at 37°C. Conversion of the bisphosphate to the 5′ monophosphate indicated the presence of pTmp (verified by UV shadowing).

### Ligation of pTmp to unmodified RNA and subsequent 3′ dephosphorylation

In a final volume of 30 μl, 1000 pmoles MH847 (unmodified RNA, sequence, see below) were incubated together with a 10× molar excess of pTmp and 1.6 U/μl RNA Ligase 1 (Thermo Scientific) in the corresponding buffer overnight at 37°C. After that, 1 U FastAP and FAP Buffer were added and the resulting solution (volume: 40 μl) was treated for 1 h at 37°C. At last, the resulting MH847pTm RNA strand was PAGE purified (see above).

### Phosphorylation of MH847pTm and MH848 (tRNA^Lys^_3_ subfragments) and modivariant synthesis

Ligation-competent MH847pTm, as well as MH848, were obtained by 5′ phosphorylation using T4-Polynucleotide kinase (see phosphorylation/dephosphorylation of RNA). The received phosphorylated RNA strands were used without further purification for the Tm modivariant synthesis (see Splint ligations using tRNA subfragments).

### Nucleic acids used for Tm mv synthesis

**Table:** 

Name	Description	Sequence (5′-3′)	supplier
MH847	tRNA^Lys^_3_ subfragment (RNA#1)	UCUGAGGGUCCAGGG	biomers
MH848	tRNA^Lys^_3_ subfragment (RNA#3)	UCAAGUCCCUGUUCGGGCGCCA	biomers

### Synthesis of permutated modivariants

Permutated modivariants were synthesized by using the RNA strands listed below. Here, RNA subfragments were 5′ phosphorylated and then subjected to splint ligations as previously described.

### Nucleic acids used for synthesis of permutated modivariants

**Table:** 

Name	Description	Sequence (5′-3′)	supplier
MH862	tRNA^Lys^_3_ subfragment (modified RNA#3)	** Cm ** UCAAGUCCCUGUUCGGGCGCCA	biomers
MH863	tRNA^Lys^_3_ subfragment (modified RNA#3)	** Am ** UCAAGUCCCUGUUCGGGCGCCA	biomers
MH864	tRNA^Lys^_3_ subfragment (modified RNA#3)	** Um ** UCAAGUCCCUGUUCGGGCGCCA	biomers
MH865	tRNA^Lys^_3_ subfragment (modified RNA#3)	** Gm ** UCAAGUCCCUGUUCGGGCGCCA	biomers
MH866	tRNA^Lys^_3_ subfragment (modified RNA#3)	** rT ** UCAAGUCCCUGUUCGGGCGCCA	IBA

### Isolation and stimulation of human immune cells

Human PBMCs were isolated from heparinized blood of healthy donors upon informed consent and approval by the local ethics committee by standard Ficoll-Hypaque density gradient centrifugation (Ficoll 1.078 g/ml). PBMCs were resuspended in complete medium prepared of RPMI 1640 (Biochrom, Berlin, Germany) supplemented with 2% heat-inactivated human serum (1 h, 56°C). For stimulation experiments, RNA was encapsulated with DOTAP (N-[1-(2, 3-dioleoyloxy)propyl]-N, N, N-trimethylammonium-205 methylsulfate) (Carl Roth GmbH Karlsruhe, Germany) at a ratio of 3 μl DOTAP per 1 μg of RNA in Opti-MEM reduced serum medium (Life Technologies) and incubation for 10 min at room temperature. DOTAP encapsulation is necessary to deliver RNA into PBMCs. For transfection experiments, cells were stimulated with RNA at final concentrations of 500, 250 and 125 ng/ml. Where indicated, methylated or unmethylated oligoribonucleotides (ORNs) and native tRNA^Lys3^ were mixed with bacterial RNA prior encapsulation with DOTAP. As a positive control, PBMCs were stimulated with bacterial RNA (see below) and TLR7/8-agonist R848 (1 μg/ml) (Invivogen, San Diego, USA). All stimulations were performed in duplicate wells per individual donor at a density of 2 × 10^5^ cells/well PBMCs in a 96-well flat bottom plate. Cells were incubated in a humidified 5% CO_2_ atmosphere at 37°C for 20 h. Cell-free supernatants were analyzed by sandwich ELISA for secretion of IFN-α (Affymetrix eBioscience, Frankfurt, Germany) according to the manufacturer’s protocol. Where indicated, values were normalized to cytokine production induced by stimulation with bacterial RNA to account for donor variation.

### Nucleic acids used for stimulation of human immune cells

**Table:** 

Name	Description	Sequence (5′-3′)	supplier
Gm18	26mer GmG - RNA	GUGGGGUUCCCGAGC ** Gm ** GCCAAAGGGA	biomers
Unmod.	26mer GG - RNA	GUGGGGUUCCCGAGCGGCCAAAGGGA	biomers

### Preparation of bacterial RNA from *Staphylococcus aureus*

*Staphylococcus aureus* ATCC 25923 was grown in Luria-Bertani (LB) Medium (Merck, Darmstadt, Deutschland) at 37°C until mid-log phase. Bacteria were harvested and treated with lysozyme (40 mg/ml, 20 min at 37°C) before RNA isolation using TRIzol reagent (Thermo Fisher, Waltham, USA) according to the manufacturer’s protocol. RNA underwent further purification using RNeasy mini kit (QIAGEN, Venlo, Netherlands) including an on-column DNase treatment. The purity and amount of bacterial RNA preparations were validated by NanoDrop measurement. Only RNA preparations with ratios 260/280 and 260/230 > 1.8 were used for stimulation.

### Statistical analysis

Data were analyzed using GraphPad Prism 6.05 (GraphPad Software Inc.). Mean plus SEM were plotted and significant differences were assessed by two-way ANOVA including multiple comparison tests or linear regression model. *P*-values are indicated by ns (not significant, *P* > 0.05), * (*P* ≤ 0.05), ** (*P* ≤ 0.01), *** (*P* ≤ 0.001). The dose-response model was calculated by R software 3.4.1 with best fitting model for the corresponding RNA. Unmodified GG ORN: log-logistic function (three parameters), native Lys3: Weibull function (two to four parameters), modified GmG ORN: log-logistic function (two parameters).

## RESULTS

### Naturally occurring RNA modifications in tRNA^Lys^_3_ decrease, but do not inhibit immunestimulation in PBMCs

As an in-depth follow-up analysis of our initial work on bacterial tRNA^Tyr^ (8) a side-by-side comparison of IFN-α secretion from PBMCs after transfection with native, fully modified tRNA^Lys^_3_ and synthesized unmodified tRNA^Lys^_3_ was performed. Bacterial RNA (bRNA) and the small molecule agonist R848 served as positive controls for TLR7 activation and non-treated PBMCs as negative control to exclude auto-activation of pDCs.

The IFN-α emission from PBMCs is a commonly used readout for TLR7 activation, which is validated by a chain of evidence, including the following elements. Depletion of pDCs from PBMCs results in complete loss of IFN-α secretion from the latter (8), and isolated pDCs show emission similar to PBMCs (not shown). Therefore the interferon response is clearly limited to pDCs. Ablation of the response upon inhibition of endosomal maturation by chloroquine treatment (8) argues for endosomal recognition and excludes cytosolic recognition. Among the TLR RNA receptors, pDCs express only TLR7 but not TLR3 or TLR8 (9) leading to IFN-α emission (1,7) as reviewed in (14,32). Of note, dendritic cells from TLR7 knockout mice did not show any response to RNA stimulation (33).

**Figure 2.**
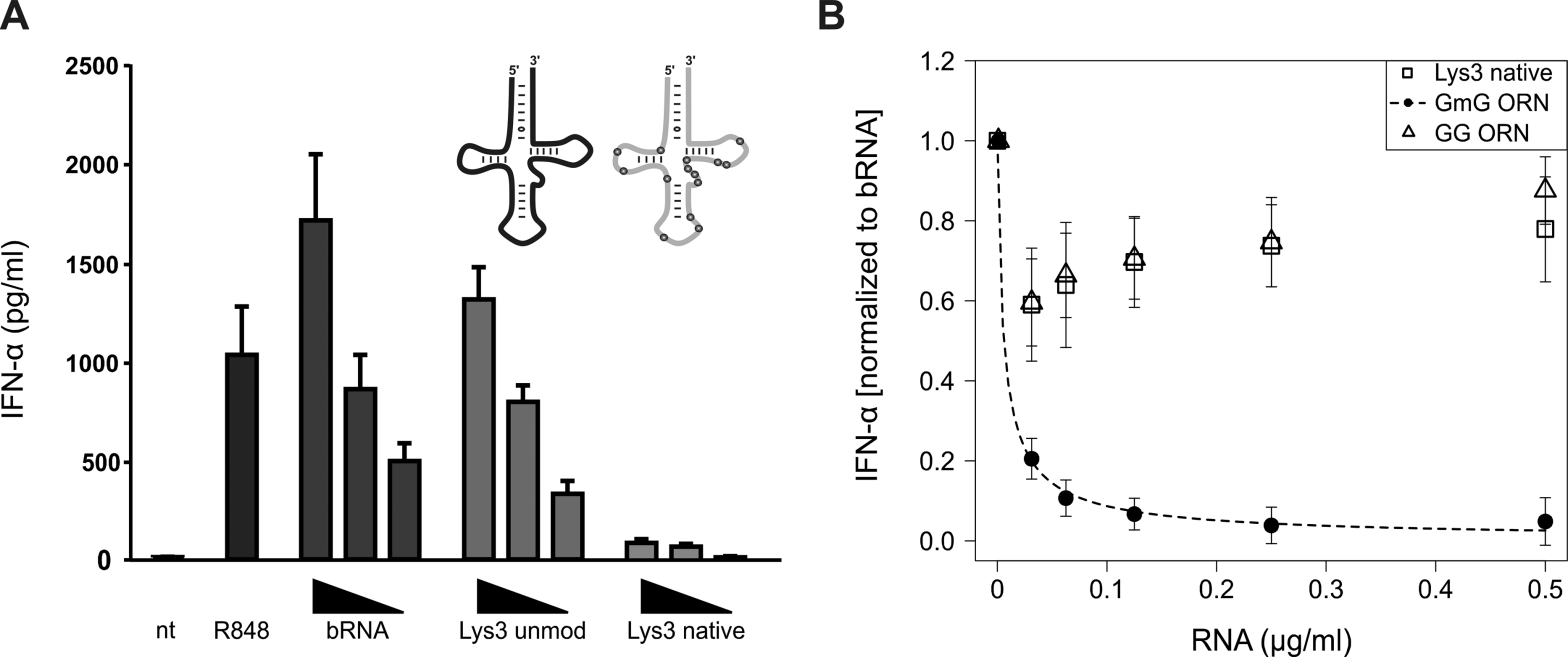
Native tRNA^Lys3^ and its immunostimulatory activity. (**A**) Human PBMCs were stimulated with DOTAP encapsulated bacterial RNA, an unmodified Lys3 tRNA and native tRNA^Lys^_3_ (see Supplementary Figure S1A) at three different concentrations (0.5, 0.25, 0.125 μg/ml). R848 (1 μg/ml) served as a positive control for TLR7 activation. IFN-α secretion was measured in cell-free supernatants after 20 h by ELISA. Shown bars represent the stimulation of 3–6 individual donors in duplicate wells +SEM. (**B**) To verify the antagonistic effect of native tRNA^Lys^_3_ human PBMCs of three individual donors were co-transfected in duplicates with a constant amount of bacterial RNA (0.5 μg/ml) and different concentrations of the indicated RNAs (0.5, 0.25, 0.125, 0.0625, 0.03125 μg/ml). Previously described GmG and GG ORNs served as positive and negative control for a *trans*-inhibitory modification, respectively. To account for donor variation data was normalized to bacterial RNA. Dose-response model was calculated by R software as described in statistical analysis.

RNA preparations were titrated in three different concentrations on isolated PBMCs. To account for donor variation, a known problem in this type of assay, three independent experiments were performed in duplicate wells. Highest IFN-α levels were observed for bRNA from *S. aureus*. A synthetic unmodified tRNA^Lys^_3_ showed nearly similar efficiency, whereas the fully modified (native) tRNA^Lys^_3_ induced only minor IFN-α levels (Figure 2A and Supplementary Figure S1A).

At first glance, these results appeared similar to those obtained from the previous comparison of native and unmodified tRNA^Tyr^ from *E. coli* (8). tRNA^Tyr^ had been identified as an antagonist for TLR7, which even inhibited immunostimulation when co-delivered in combination with an otherwise immunostimulatory tRNA. To investigate the immunomodulating properties of tRNA^Lys^_3_ in more detail and in particular to study, whether tRNA^Lys^_3_ exerts inhibitory effects as observed for tRNA^Tyr^, we designed an assay to discriminate between immunosilencing and antagonistic abilities. In this assay, changes of the IFN-α secretion in response to constant amounts of immunostimulating bRNA combined with different concentrations of tRNA^Lys^_3_ were measured. As a positive control for TLR7 inhibition, the combination of bRNA with a Gm-containing 26mer (GmG ORN, mimicking the inhibitory activity of tRNA^Tyr^) was analyzed: whereas pure bRNA induced IFN-α secretion, the addition of the GmG ORN containing a single modified Gm led to complete suppression of TLR7 activation even at moderate concentrations (Figure 2B). As specificity control for inhibition of TLR7, we used an unmodified 26mer (GG ORN) which did not decrease bRNA induced IFN-α levels. Further details of the assay validation were previously published in Gehrig *et al.* (8) and Schmitt *et al.* (17).

Importantly, the addition of tRNA^Lys^_3_ did not decrease IFN-α levels induced by bRNA, indicating that the immune-modulatory effects of modifications within tRNA^Lys^_3_ were only sufficient to prevent TLR7 activation in *cis* (Figure 2A) but not acting inhibitory in *trans* to another RNA (Figure 2B). These findings demonstrate that tRNA^Lys^_3_ is non-stimulatory, though not acting as a TLR7 antagonist and thus differs from the previously characterized Gm18 modified tRNAs with respect to modulation of TLR7. Given that unmodified tRNA^Lys^_3_ is fully stimulatory, the modified nucleotides in tRNA^Lys^_3_ (Figure 1) are obvious mediators of the non-stimulatory (*cis*-silencing) effect.

### Molecular surgery employing the tRNase Colicin D reveals reduced immunostimulation of the 3′ part of native tRNA^Lys^_3_

To examine the immunomodulatory contribution of post-transcriptional modifications in native tRNA^Lys^_3_, we synthesized tRNA^Lys^_3_ -modivariants using a molecular surgery procedure (8). Modivariants are RNA species that are identical in sequence but differ in post-transcriptional modifications. Their comparative testing allows to conclude on the influence on TLR7-mediated immunostimulation of a single or a subset of modifications. Modivariants can be synthesized by joining modified and unmodified fragments using biochemical methods that are frequently described as ‘cut-and-paste’ or ‘molecular surgery’ techniques (9,32,34). We have previously described the generation of RNA fragments from tRNAs that were well defined in terms of length and modification content by a cleavage reaction mediated by catalytic DNA, so-called DNAzymes (8,35). While the cleavage target site of DNAzymes can be programmed (23), their cleavage efficiency turned out to be impaired by the presence of multiple RNA modifications, which is a typical situation for eukaryotic tRNAs.

**Figure 3.**
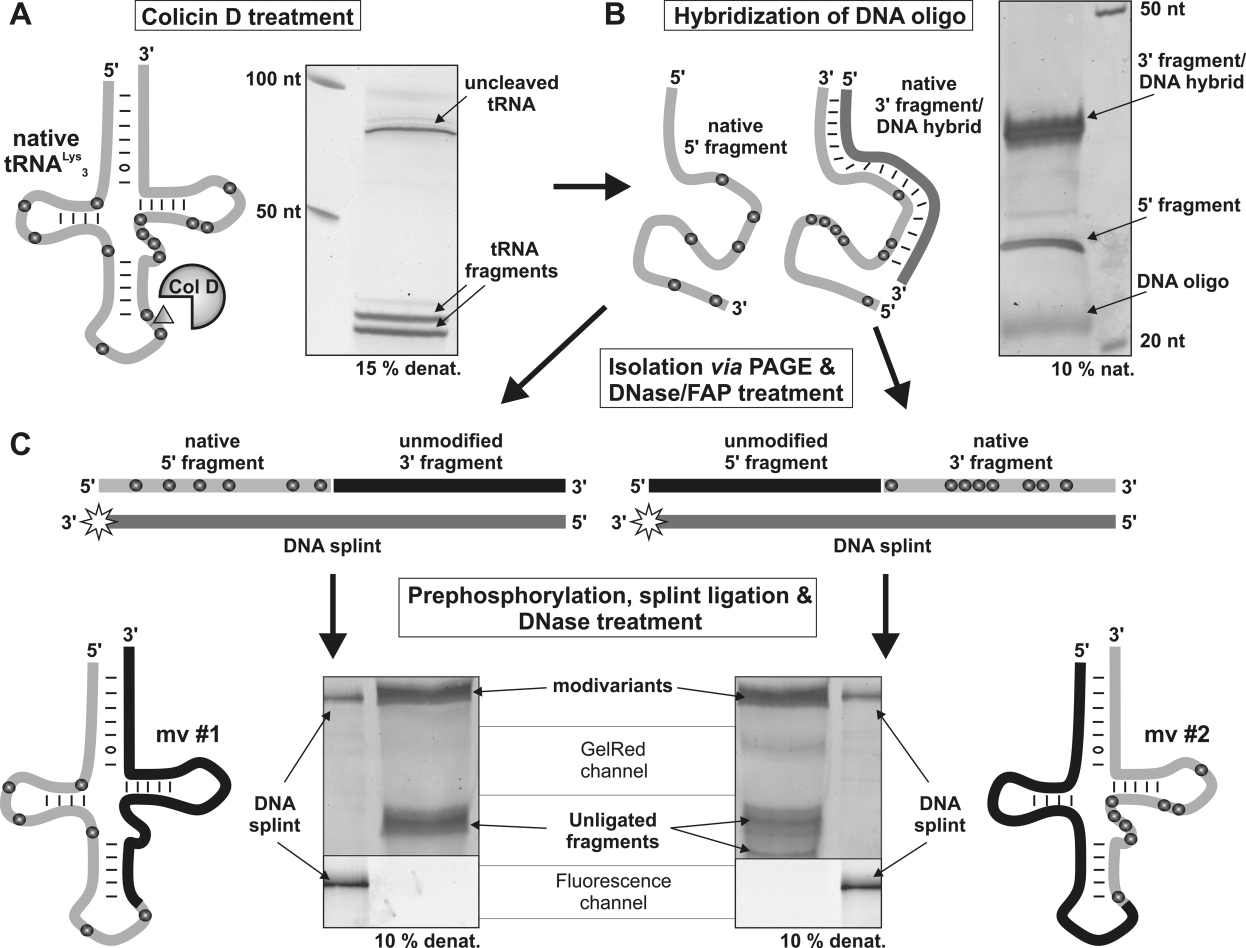
Colicin D-based molecular surgery and preparation of Lys3-modivariants. (**A**) Treatment of native tRNA^Lys^_3_ (gray line, modifications indicated as gray dots) with Colicin D generated two fragments with 38 nt length (see PAGE analysis). Differences in migration distance were caused by 2′-3′-cyclic phosphates at the 3′ end of the 5′ fragment. (**B**) Due to similar fragment lengths, a DNA oligonucleotide (darker gray) was hybridized to the 3′ fragment. The corresponding band shift of the double-stranded RNA/DNA hybrid enabled separation and isolation of both tRNA fragments. RNAs were then treated with DNaseI and FastAP. (**C**) Prephosphorylated 5′ and 3′ fragments were subsequently annealed together with unmodified RNAs (black lines) on splint DNA (indicated by the darker gray line, fluorescence marker displayed as a star) which contains a 3′ fluorescein residue. Ligation and DNase treatment yielded mv#1 and mv#2 as shown in the respective PAGE analysis. Evaluation of the fluorescence channel revealed successful digestion of DNA splint in modivariant preparations.

We therefore turned to an alternative approach, making use of the enzymatic activity of Colicin D (28). This tRNase was reported to specifically cleave tRNA^Arg^ isoacceptors between positions 38 and 39 in the anticodon-loop (28) *in vivo*. Interestingly, we observed that fully modified mammalian tRNA^Lys^_3_ was also a target of Colicin D cleavage *in vitro*, which resulted in two well-defined tRNA fragments (Figure 3A). Of note, the cleavage between position 38 and 39 resulted in two RNA fragments of similar size, leading to separation problems by denaturing PAGE. To address this issue, we hybridized a complementary DNA oligonucleotide to the cleaved 3′ fragment. On a native PAGE, the resulting DNA/RNA hybrid (Figure 3B) was considerably less mobile than the 5′-fragment. This procedure enabled simultaneous separation and isolation of both fragments, which were then submitted to treatment with DNase and alkaline phosphatase, followed by phenol–chloroform extraction. Subsequently, the corresponding RNAs were treated with T4-PNK and ATP to remove any cyclic 3′-phosphates and to obtain 5′-phosphorylated, ligation-competent strands (Figure 3C). Native tRNA fragments were then combined with equimolar amounts of a 5′-phosphorylated, synthetic, unmodified RNA, and hybridized to a full-length cDNA to yield a ligation-competent complex. These nicked DNA–RNA hybrids served as substrates for T4-DNA ligase, and after ligation, the DNA splint was removed enzymatically, with typical isolated yields between 40–60% (36). At last, both obtained modivariants were purified *via* denaturating PAGE and subjected to immunostimulation tests with PBMCs.

**Figure 4.**
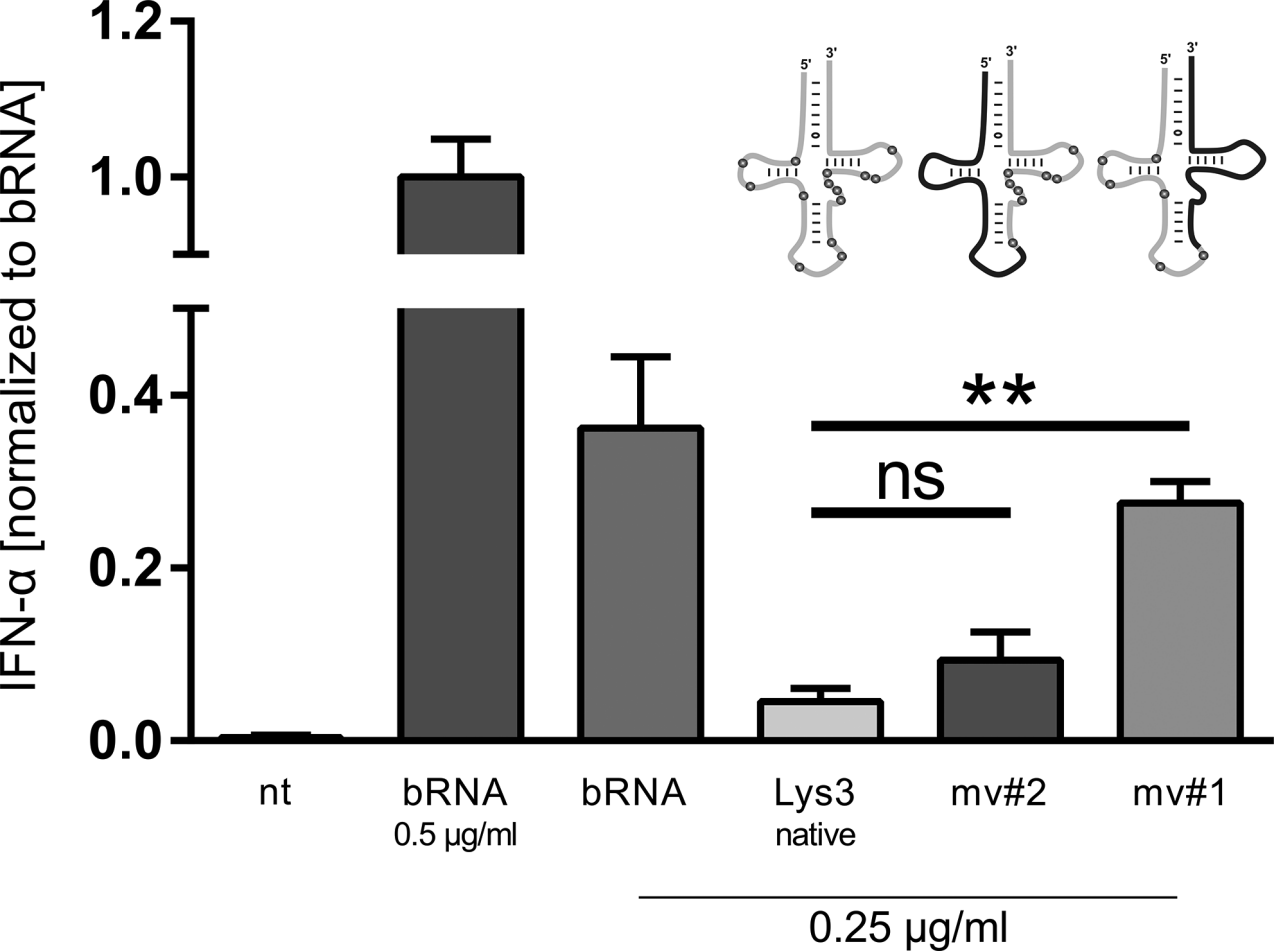
Immunostimulatory activity tests of Lys3-modivariants. Human PBMCs were stimulated with DOTAP encapsulated RNA species at indicated concentrations. Splint-ligated mv#1 and mv#2 containing half part of native tRNA^Lys^_3_ and unmodified RNAs as well as native tRNA^Lys^_3_ were tested on three individual donors in duplicate wells. IFN-α levels were measured in cell-free supernatants 20 h post-transfection. To account for donor variation results were normalized to the amount of IFN-α induced by 0.5 μg/ml bRNA. Each bar illustrates mean +SEM.

As depicted in Figure 4, bacterial RNA and modivariant 1 (mv#1, which included the native 5′ fragment and its modifications) induced comparable IFN-α secretion, indicating that none of the modifications in the 5′ fragment was sufficient to abolish TLR7 stimulation. In contrast, fully modified tRNA^Lys^_3_ and modivariant 2 (mv#2, which contains the native 3′ fragment) displayed clearly reduced immunostimulatory activity. Taken together, these results indicate that a single modified residue or the combination of multiple modifications in the 3′ part of native tRNA^Lys^_3_ caused decreased immunostimulation.

### Tm-modivariant displays decreased immunostimulatory activity

Based on this latter observation, we further inspected the underlying sequence context of the 3′ part of tRNA^Lys^_3_, which harbors the following RNA modifications (depicted in Figure 1): Ψ39, m^7^G46, D47, m^5^C48, m^5^C49, Tm54, Ψ55 and m^1^A58. So far, none of these modifications has been associated with reduced PBMC-stimulation when present in tRNAs. Yet, synthetic RNAs containing high amounts of Ψ and m^5^C exhibited low immunostimulation toward TLR7 and other RNA sensors (21,37,38). Bacterial tRNAs containing Ψ, m^7^G, D, t^6^A or T were stimulatory toward TLR7 (8), as was yeast tRNA^Asp^ which contains m^5^C (not shown).

Among the modifications present in the mv#2, we focused on Tm which, in addition to the ubiquitous T (m^5^U) modification, also featured a 2′-O-methylated ribose moiety. The latter also occurs in Gm, which had been described as an inhibitory modification in recent studies (8,18). However, in various preceding permutation studies, inhibition had remained restricted to ribose- methylated purines, while the corresponding pyrimidines were innocuous.

**Figure 5.**
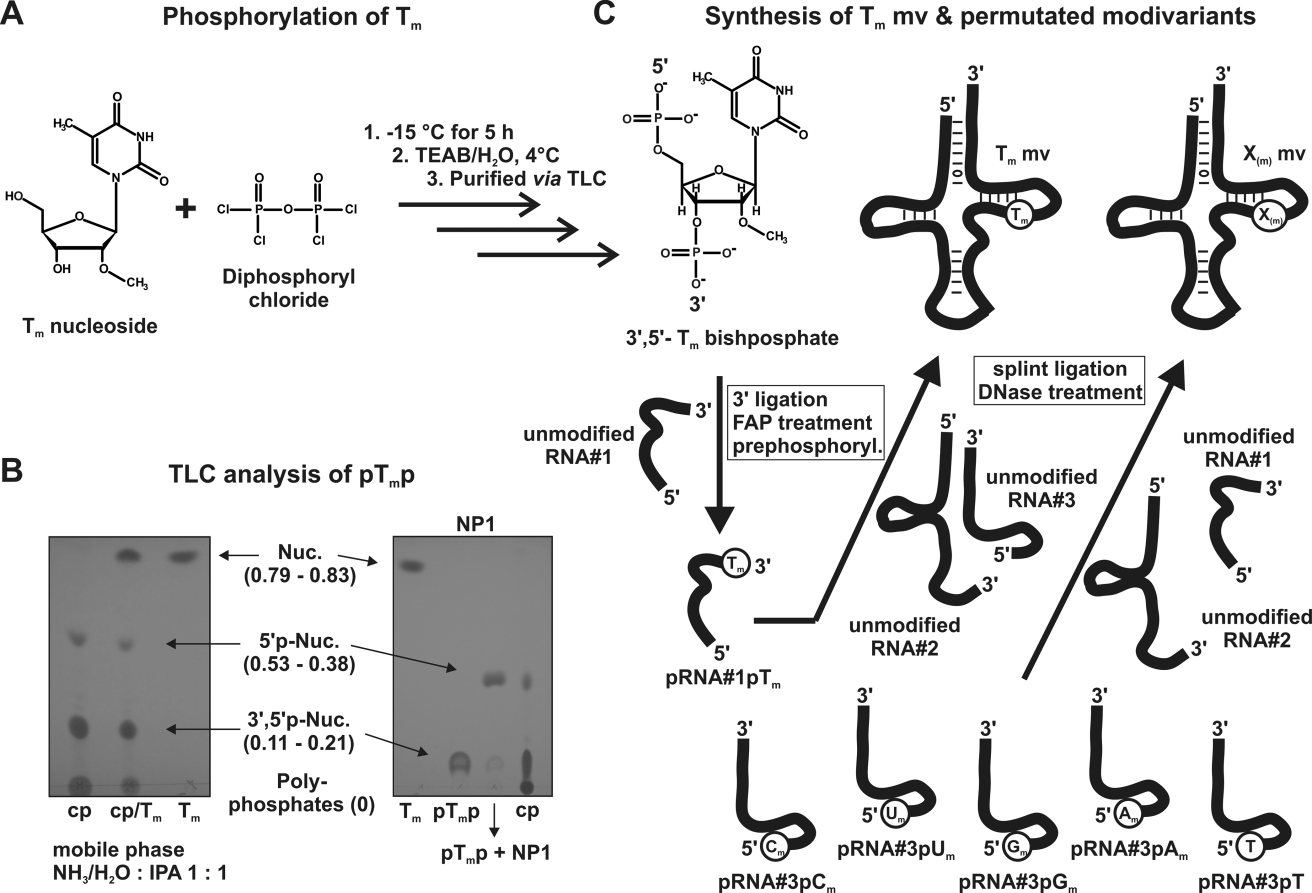
Synthesis of Tm mv & permutated modivariants. (**A**) Reaction scheme describing the conversion of Tm to the corresponding bisphosphate by diphosphoryl chloride addition (**B**) Left part: TLC analysis of crude product (cp) showed the formation of 5′-monophosphate, 3′-5′-bisphosphate and polyphosphates (according to (31)). The Tm nucleoside (Tm) was not detectable which indicated complete conversion. Co-spotting (cp/Tm) of crude product and nucleoside is displayed in the middle lane. Right part: Isolated bisphosphate (pTmp) was treated with nuclease P1 (pTmp + NP1). The changed Rf value revealed the formation of the 5′-monophosphate out of the bisphosphate which proved pTmp-synthesis. (**C**) For the synthesis of the Tm modivariant, the respective bisphosphate was attached to an unmodified RNA#1 (part of tRNA^Lys^_3_) using RNA ligase 1. Further reaction steps then yielded the ligation-competent and Tm-modified RNA#1 which was subsequently ligated to RNAs #2 and #3 producing the desired modivariant. Xm modivariants were created the same way using commercially synthesized RNAs which were already attached to X(m) modifications.

Thus, we synthesized a tRNA^Lys^_3_-Tm modivariant containing Tm at position 54 in an otherwise unmodified tRNA^Lys^_3_ context. The first step of this synthesis included the bisphosphorylation of a commercially available Tm nucleoside with diphosphoryl chloride (Figure 5A). Successful preparation of the bisphosphorylated nucleoside (pTmp) was verified by thin-layer chromatography and nuclease P1 conversion of the bisphosphate to the 5′-monophosphate (Figure 5B). According to literature (30), nucleoside bisphosphates are minimal substrates for T4 RNA ligase 1, which selectively catalyzes the attachment of 5′,3′-bisphosphates to the 3′ end of single-stranded RNAs. Accordingly, pTmp was ligated to a synthetic unmodified RNA fragment (RNA#1) of tRNA^Lys^_3_ (Figure 5C) with about 25% isolated yield. After subsequent 3′-dephosphorylation and 5′-prephosphorylation by T4-PNK, pRNA#1pTm was used in a splint ligation together with the remaining RNA strands (#2 and #3) to yield the desired Lys3-Tm modivariant (Supplementary Figures S1B and C). Isolated yields were typically 15–25%, with ∼60% ligation efficiency per ligation site and some 30–50% loss during isolation of the final RNA constructs (36).

The presence of Tm within the modivariant was verified via LC-MS/MS analysis (see supplementary information). As displayed in the corresponding diagram (Supplementary Figure S2), each modivariant molecule contained one Tm residue. The correct position of the Tm modification was furthermore confirmed by RiboMethSeq (39) (results are shown in Supplementary Figure S3).

**Figure 6.**
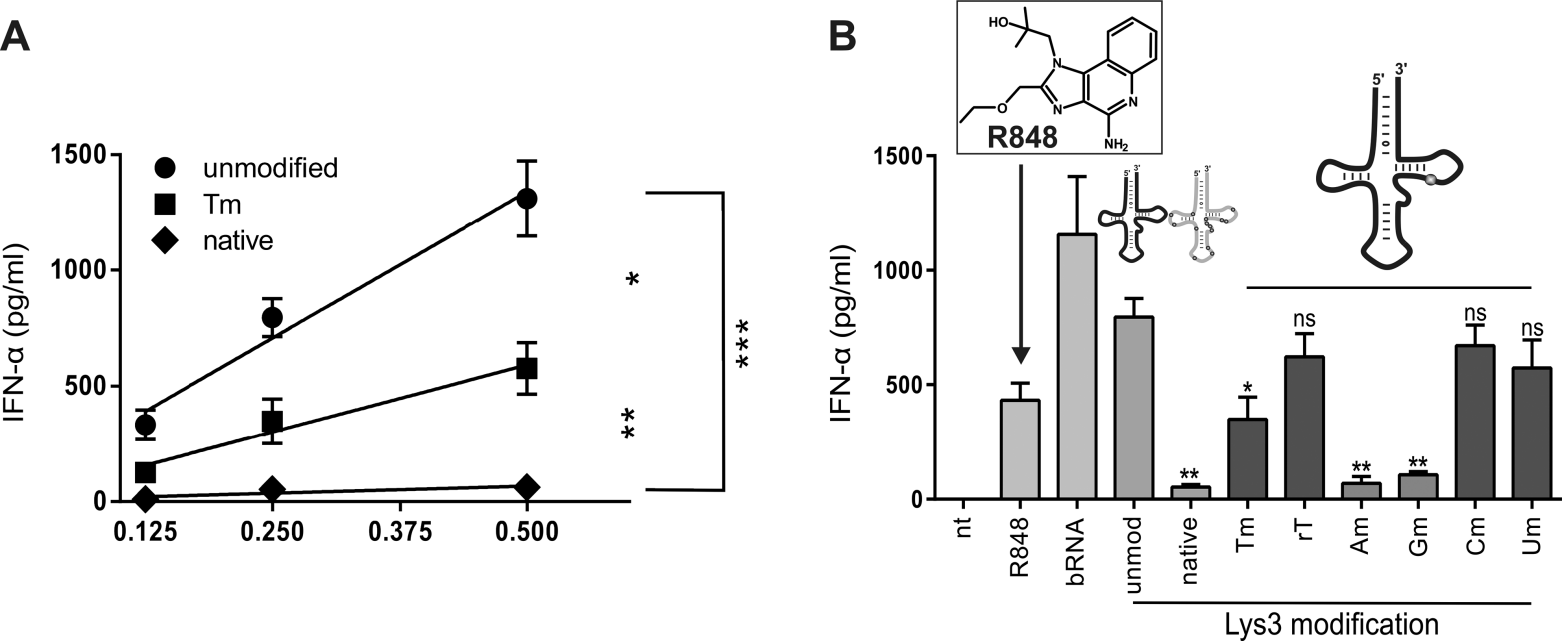
Immunostimulation of single modified tRNAs. (**A**) Isolated PBMCs were stimulated with indicated tRNAs encapsulated with DOTAP at a concentration of 0.5, 0.25 and 0.125 μg/ml. Cell-free supernatants were collected after 20 h and IFN-α release was detected by ELISA. Linear regression was calculated by GraphPad Prism. (**B**) Transfection of single modified tRNAs incorporating indicated modifications at position 54 of tRNA sequence at a final concentration of 0.25 μg/ml. R848 and bRNA were used as a positive control for TLR7 stimulation. IFN-α secretion was compared to unmodified tRNA control. All stimulations were performed on three individual donors in duplicate wells. Shown are mean + SEM.

The obtained modivariant was subsequently tested in comparison to the fully modified tRNA^Lys^_3_, which itself showed hardly any stimulation, and in comparison to a completely unmodified modivariant of tRNA^Lys^_3_. The introduction of the Tm modification efficiently reduced the immunostimulatory activity of the otherwise unmodified full-length tRNA by almost 50%. Of note, the native tRNA^Lys^_3_, was even less stimulatory than the Tm modivariant, indicating an immunosilencing effect of further modifications (Figure 6A).

### Analysis of Xm-modivariants suggests a synergistic effect of nucleobase- and ribose-modifications on IFN-α secretion

To better understand details of Tm function in the context of tRNA^Lys^_3_, we permutated the nucleobase at position 54 as previously described (18). The starting material for these permutations were commercially available RNA oligonucleotides with 2′-O-methylcytidine (Cm), 2′-O-methyluridine (Um), 2′-O-methyladenosine (Am) or 2′-O-methylguanosine (Gm) at their 5′ ends. This allowed straightforward splint ligations utilizing the remaining unmodified RNAs #1 and #2 (see above and Supplementary Figures S1B and C).

Subsequent immunostimulation tests of these mutant/modivariants demonstrated significant differences between 2′-O-methylated purines and pyrimidines (Figure 6B). The modified pyrimidines Cm and Um induced IFN-α levels comparable to unmodified tRNAs. In contrast, the purine derivatives Am and Gm decreased the immunostimulatory activity of RNAs to levels which were comparable to those of native tRNA^Lys^_3_.

As a further observation of considerable interest, the presence of ribothymidine (T, rT) without ribose methylation did only slightly affect stimulation, similar to Um (Figure 6B). While single methylation neither of the ribose nor of the base of U54 was at all effective in decreasing the stimulation of TLR7, the combination of both showed a strong synergy, amounting to the observed 50% decrease in comparison to unmodified tRNA^Lys^_3_. The above observations may have implications for the recognition mode by TLR7, namely that Tm presumably binds to the same site and in a similar orientation as do Gm and Am.

As a final observation, this remarkable synergistic effect of Tm within tRNA^Lys^_3_ can only partially explain the observed low stimulatory potential on PBMC. Indeed, our results also imply a contribution of the remainder of the modifications shown in Figure 1, either single or as an ensemble.

## DISCUSSION

### A new molecular tool for biochemical manipulation of RNA *in vitro*

As indicated by our recent studies (8), ‘self’ tRNA is guarded against TLR7 activation by RNA modifications, such as Gm18, Gm34 and one or more hitherto unknown modifications in other tRNAs, including in particular tRNA^Lys^_3_, the object of the present work. Given that eukaryotic tRNAs may contain up to 25% modified nucleotides (40), the need to create modivariants containing selected defined modifications became even more imperative than in previous studies of bacterial tRNA, which are generally less modified (40). However, it turned out that the very same modifications impede our previously used DNAzyme approach for site-specific tRNA cleavage. The subsequent development of Colicin D, a ribotoxin with tRNase activity (28), as a new tool in the repertoire of methods often subsumed as ‘molecular surgery’ or ‘cut-and-paste’ of RNA biochemistry is an added bonus. Indeed, we anticipate its continued application in further studies of eukaryotic tRNAs, since preliminary data indicate, that the restriction of tRNase activity of Colicin D to tRNA^Arg^ and tRNA^Lys^ can be overcome by increasing enzyme levels. So far, we succeded in conducting controlled cleavage of a yeast tRNA^Phe^ transcript. Also, yeast total tRNA could be digested to >90%, leaving significant amounts of defined ∼30mer tRNA fragments (data not shown), suggesting that *in vitro* conditions for controlled cleavage of for any tRNA shaped molecule can be developed.

### Distinction between minor and major determinants of immune suppression

Colicin D-based molecular surgery gave access to two modivariants whose testing identified a major effect caused by the modifications situated in the 3′-part. Among those modifications present, several occur in bacterial and other microbial tRNAs that had previously been found to be stimulatory. These modifications were considered as highly unlikely candidates for the observed effect.

The remainder consist of m^1^A58 and Tm54. Given the previous findings on ribose-methylated modifications, we turned our attention to the synthesis of a modivariant containing Tm54 as sole modified nucleotide. In this context, Tm decreased the interferon response by 50%. While this is a pronounced effect, it is considerably weaker than what was typically observed for Gm. Of note, since the fully modified tRNA^Lys^_3_ evokes almost no response, it is clear that the remainder of modifications, i.e. the ensemble except Tm, also has a contributing effect. Further studies may identify additional contributions of single modifications, but we would like to point out some of our results with synthetic modifications in this context. We recently reported a generic ‘shielding’ effect of a number of synthetic RNA modifications with no discernible common denominator in terms of chemical structure (41). The conclusion from those studies might also apply to heavily modified eukaryotic tRNA. An RNA carrying multiple and especially bulky modifications might escape detection by an altogether altered set of functional groups that mask the presence of typical RNA features. In the case at hand, the anticodon of tRNA^Lys^_3_ would be shielded by two bulky modifications (ms^2^t^6^A and mcm^5^s^2^U). Furthermore, modifications in the anticodon stem as well as in the D-loop are so numerous that they approach a density previously investigated in *in vitro* transcripts, which contained high levels of, e.g. Ψ or m^5^C (21,37,38). With regard to a fast immune response toward foreign RNA, the incorporation of bulky, and therefore likely unstimulatory, but not inhibitory modifications within human tRNAs may be crucial. Antagonistic effects of self-RNA would block the receptor and therefore impede an adequate immune response against invading pathogens.

### Tm as a major determinant of immunosilencing

Our results indicate that Tm at position 54 is responsible for much of the interesting effect observed in tRNA^Lys^_3_. Contrary to Gm18, tRNA^Lys^_3_ reveals no dominant inhibitory effect when delivered in *trans* with a stimulatory RNA. In fact, our findings imply some kind of ‘RNA muting’ and the data indicate that some 50% of this ‘muting’ is due to the presence of Tm. As already discussed, this implies that other modifications within the RNA are also necessary for the full effect of tRNA^Lys^_3_. Of note, Tm therefore classifies differently from previously identified immune-modulatory Gm and Am modifications, the latter also suppressing in *trans*. In a previous study, we identified a minimal tri-nucleotide motif within RNA which is necessary and sufficient to antagonize TLR7 and TLR8 (17). A key finding of this study was the relevance of the nucleobase downstream the 2′-O-methylated nucleotide for immunosuppression. In case of the native tRNA^Lys^_3_, Tm is followed by pseudouridine whereas in the described modivariants Xm was followed by a uridine. The effect of Tm might potentially be modulated by synergistic effects with further modifications in the defined sequence context of the native tRNA.

TLR7 reportedly responds strongly to single-stranded RNA rich in guanosine and uridine residues (14). Supported by x-ray structures of TLR7 and TLR8 (12,13), recent reports postulated separate binding sites for both types of nucleosides in murine TLR7. Furthermore, RNA hydrolysis was discussed as an intrinsic part of TLR-signalling (12,42). In this backdrop, the current results raise multiple questions. Importantly, we are primarily assessing the evasion/inhibition of TLR7 signaling, rather than the signaling itself. It is therefore not at all clear if Tm and Gm mediate their TLR7-silencing effect through the same binding site that mediates TLR7-activation by U and G residues. It is furthermore open if immunesilencing by ribose-methylated nucleotides requires binding of RNA to one, two or even yet additional sites not yet identified by structural biology. Own data suggest that Gm modifications act as competitive antagonist inhibiting signaling by stimulatory RNA (24).

### Progress toward a pharmacophore for TLR7 inhibition?

**Figure 7.**
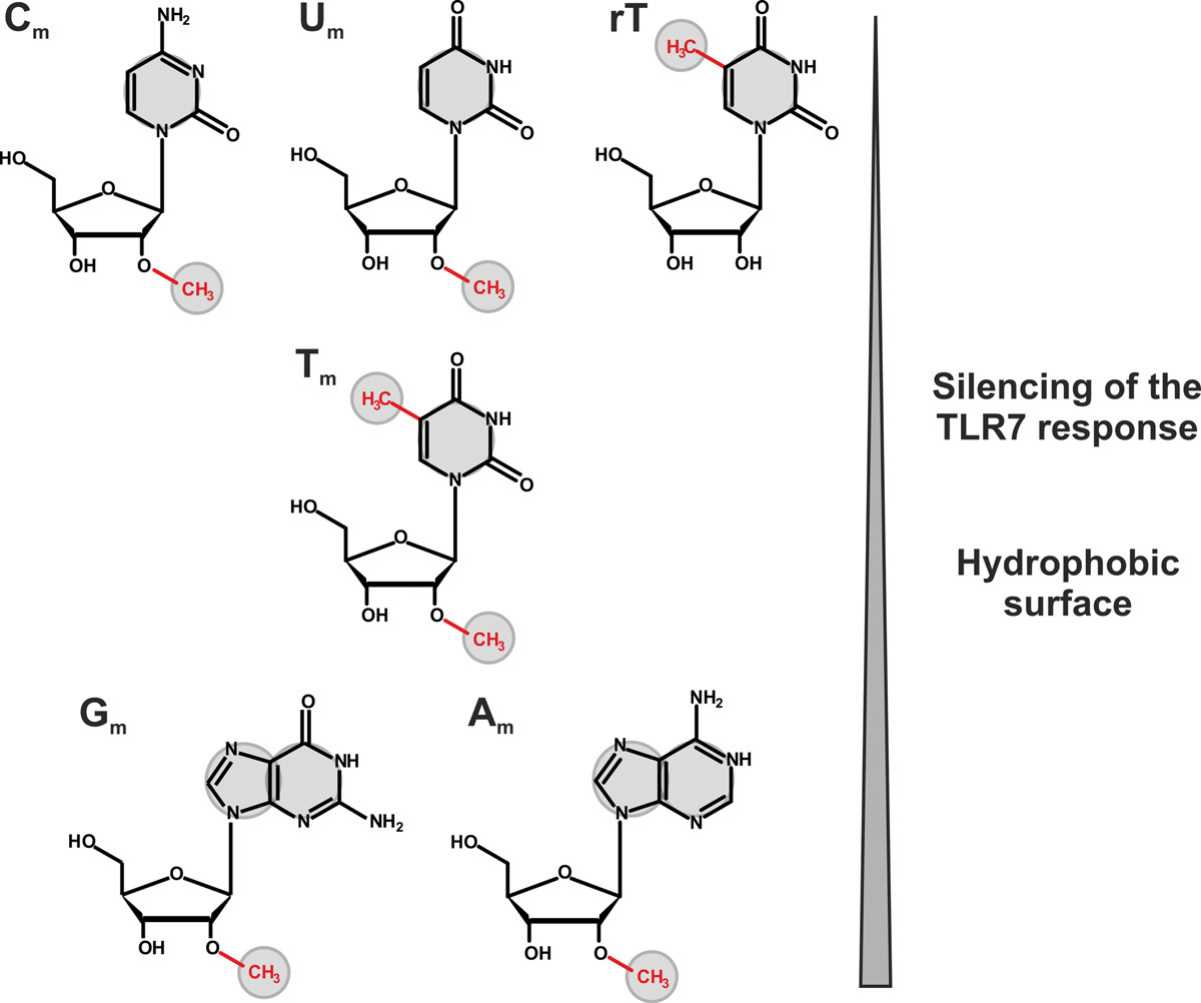
Correlation between the hydrophobic surface of RNA modifications and their immunosilencing potential. Modifications in the upper part possess only small hydrophobic surfaces (indicated by gray circles) in comparison to purine compounds (Gm and Am). The latter also reveal the highest immunosilencing potential.

Pure biochemical data of the type presented here cannot unambiguously determine the number and structure of effective binding sites. However, we have added an interesting piece of data by comparing ribose-modified nucleosides Cm, Um, Tm, Am and Gm at the precisely same position of a tRNA scaffold (n.b.: the effects of Am and Gm do not differ significantly in Figure 6B, but previous studies showed Gm to be more potent in detailed dose-titration studies (17)). Furthermore, the sequence context in which the 2′-O-methylated nucleobase occurs is crucial for the antagonistic effect of an RNA. If Gm is followed by cytidine, no dominant inhibitory effect is detectable. Immunostimulation decreases from Cm to Gm, suggesting that the binding site is suited to optimally recognize features of a purine residue, and of course the 2′-O-methyl group. The synergistic effect of the two post-transcriptional methylations resulting in Tm thereby creates structure bearing similarities to Gm. These similarities concern the hydrogen pattern on the Watson–Crick face, but more importantly, the C5-methylation increases the hydrophobic surface of the base, bringing it closer to that of a purine ring (Figure 7). We therefore speculate that Tm mediates its effect by binding to the same site as Gm, using a hydrophobic interaction, potentially π-stacking, in addition to hydrogen bridges involving O6G/O4U as donor and NH1G/NH3U as acceptors. This view is supported by a recent study on the related TLR8, where stimulation was efficiently inhibited by a 7-deaza-8-azainosine structure, a purine whose hydrogen pattern on the Watson–crick face shows common features with the above (43). This study also indicates a direction for follow-up research on the present topic.

### Biology of the Tm modification

Several lines of evidence disencourage the notion, that unmodifed tRNAs as used in this study, are recognized because of their failure to properly fold into the typical threedimensional L-shape of tRNAs. In particular, unmodified yeast tRNA^Phe^ and mammalian tRNA^Lys^_3_ had previously been subject to structural comparison with their fully modified counterparts (44–47). Based on the minor differences reported, Tm might have subtle impact on local structure at best, making impact on TLR-recognition implausible. Also, in previous unpublished experiments, we found some TLR7 inhibtion by Gm in fragments of tRNA^Tyr^, and similarly, low TLR7 response for Tm containing fragments compared to unmodified fragments. These observations argue for the overall tRNA structure to be irrelevant in TLR/ recognition. Indeed, the concept that TLR7 inspects small unstructured stretches of RNA is supported by TLR7 inhibtion of Gm-containing RNA oligomers as short as 9 nt (17).

From the biological point of view, it might be possible that Tm serves as one of several modifications that differentiate between foreign- and self-RNAs. Whereas ample literature is available concerning the T54 modification (10,48), little is known concerning Tm beyond its identification (40,49). In the threedimensional structure of a canonical tRNA, T54/Tm54 are engaged in tertiary interactions that involve also G18/Gm18, and both residues are thus in close spatial proximity (10,48,49). So far, no tRNAs have been reported to simultaneously contain both Gm and Tm (40). Interestingly, the ensemble of modifications in tRNA^Lys^_3_ (Figure 1) is involved in HIV replication in multiple ways. For example, mcm^5^s^2^U34 is involved in the annealing process of the tRNA as primer for HIV reverse transcriptase (47,50), and m^1^A58 causes an arrest of reverse transcription that is mandatory for HIV replication (51). Given that tRNA^Lys^_3_ is selectively packaged into the HIV-virion (52,53), the presence of Tm in this tRNA might contribute to lowering an interferon response, although a tRNA containing Gm would arguably have a stronger effect. Of note, it was reported that pDCs of chronically HIV-infected patients displayed a reduced IFN production on a per-cell basis after treatment with TLR7 agonists (54). However, at this point, further interpretation is to be approached with great caution.

Part of our future research will be aimed at the contribution to immunoevasion of modifications other than Tm. We note that, while tRNA modifications in *E. coli*, yeast and mammalian mitochondria (40,55) have been mapped with high precision, knowledge on mammalian cytosolic tRNA modification patterns remains very sparse, and this constitutes a significant drawback in this extremely vibrant field, which currently develops intrinsic connections to medicinal biology.

## Supplementary Material

Supplementary Data
