# Supplementary material for: Double methylation of tRNA-U54 to 2′-O-methylthymidine (Tm) synergistically decreases immune response by Toll-like receptor 7
Source: Nucleic Acids Res. 2018 Aug 8;46(18):9764–75. doi: 10.1093/nar/gky644 (PMC6182150; doi:10.1093/nar/gky644)
Supplement: Supplementary Data [file gky644_supplemental_files.doc]

**Supplementary information**

**Synthesis and purification of the Lys3 modivariants**


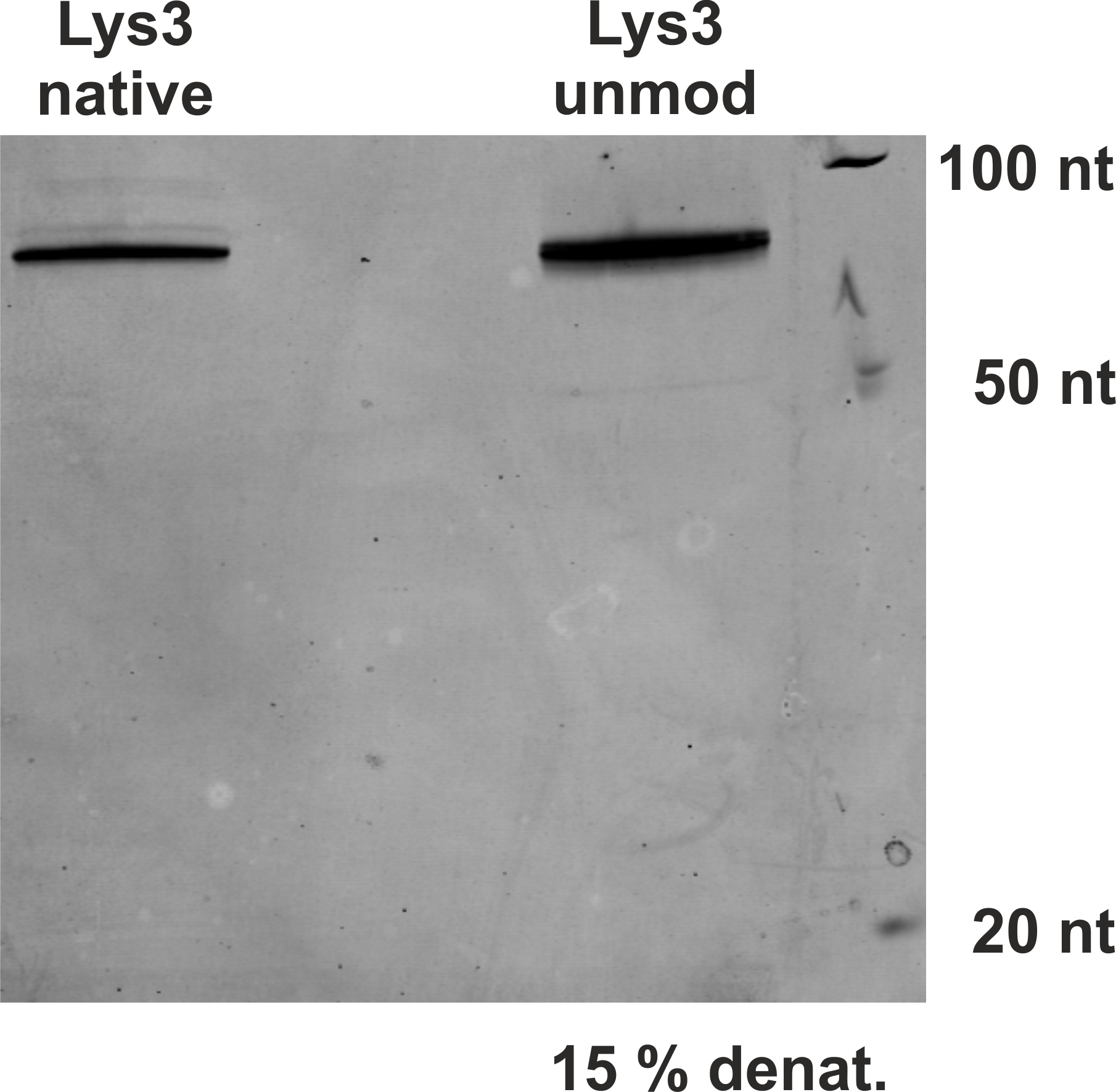
**A**

**
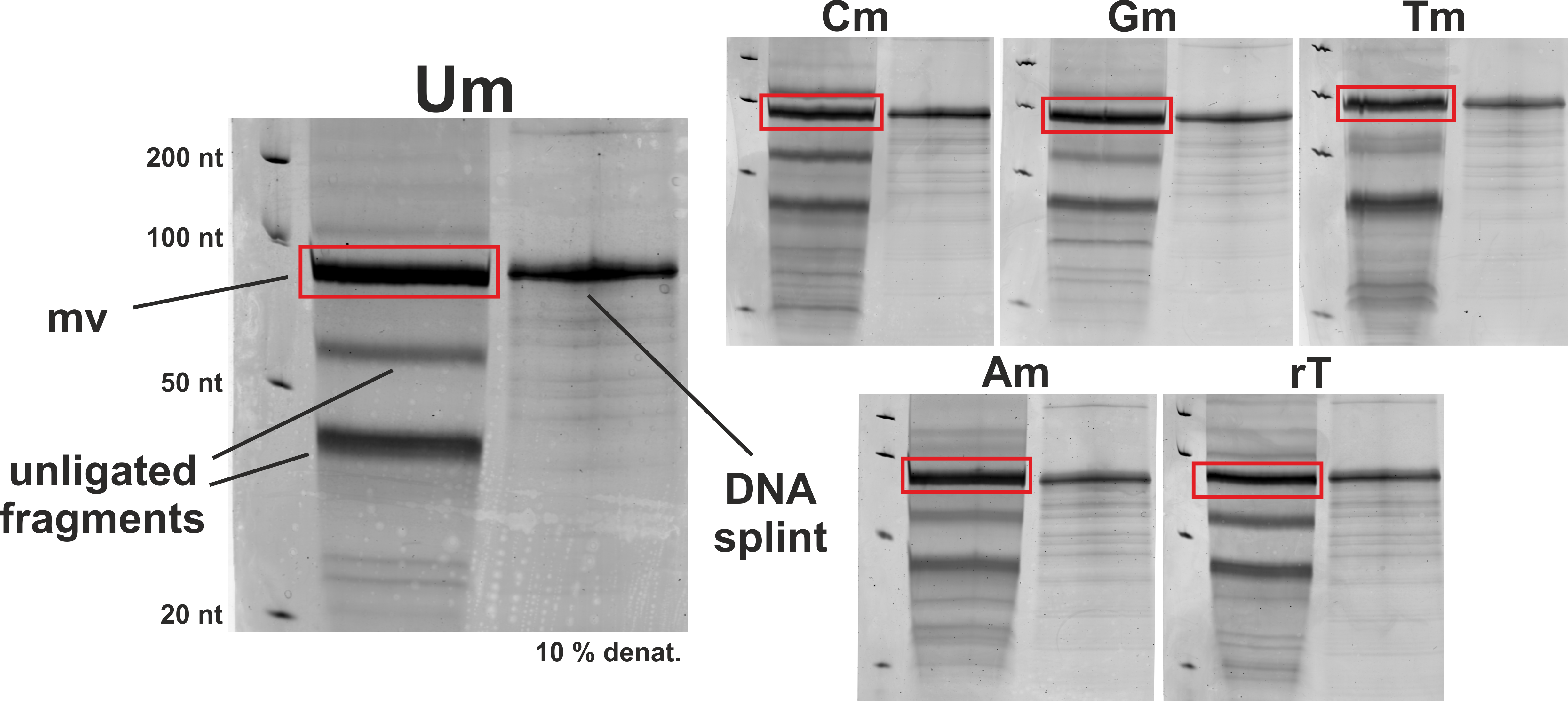
**

**B**

**C**

**
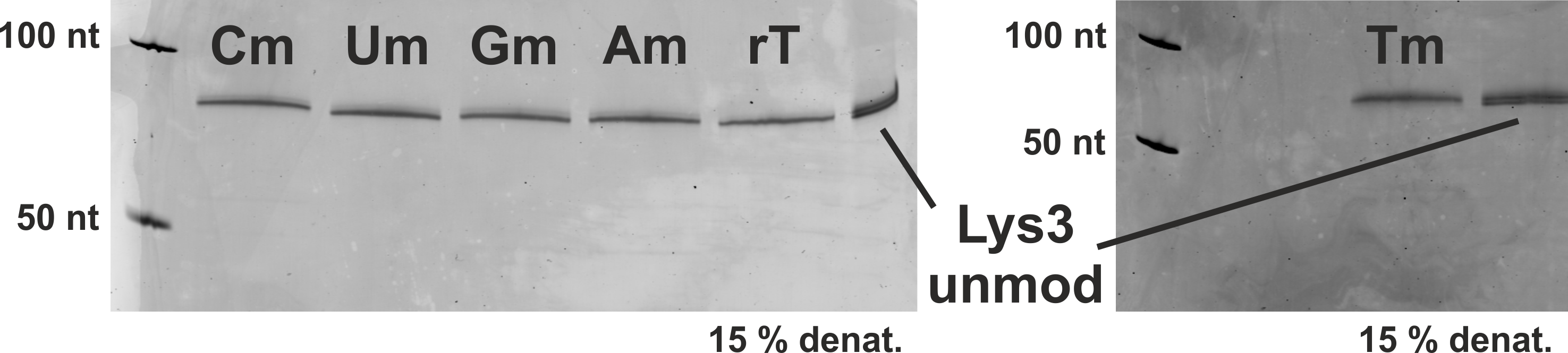
**

**Figure S1. PAGE analysis of investigated Lys3 RNA samples.** (A) The gel picture shows a denatured PAGE analysis of the unmodified (Lys3 unmod) and modified tRNA^Lys^_3_ (Lys3 native) sample used for immune stimulation (see Fig. 2 A). (B) Preparative PAGE gels displaying the synthesized modivariants (‘mv’, red boxes) and unligated fragments. (C) Gel pictures of the isolated/purified modivariants and an unmodified Lys3 tRNA (size control).

**LC-MS/MS analysis of Tm modivariant**

The Tm modivariant was digested to the nucleoside level as it was described before (1):

420 ng RNA, 0.3 U nuclease P1 (Sigma Aldrich, Germany), 0.1 U snake venom phosphodiesterase (Worthington, USA), 1/10 vol. of 10x nuclease P1 buffer (225 mM NH_4_OAc pH 5.0, 0.2 mM ZnCl_2_), 500 ng Tetrahydrouridine (cytidine deaminase inhibitor, Merck) and 200 ng Pentostatin (adenosine deaminase inhibitor, Sigma Aldrich) were incubated together for 2 h at 37 °C. Afterwards, 1 U FastAP and 1/10 vol. of 10x FastAP buffer were added followed by another incubation at 37 °C for 1 h. For the subsequent LC-MS/MS analysis 10 ng of digested modivariant were employed (measured in technical triplicates).

The nucleoside mixture was analyzed using an Agilent1260 series equipped with a diode array detector (DAD) and a Triple Quadrupole mass spectrometer (Agilent 6460) with an electrospray ion source (Agilent Jet Stream). In addition, a Synergy Fusion RP C18 column (4 µm particle size, 80 Å pore size, 250 mm length, 2 mm inner diameter) from Phenomenex (Germany) was utilized at 35°C. 5 mM ammonium acetate buffer (pH 5.3) was used as solvent A and acetonitrile (LCMS grade, Sigma Aldrich) was used as solvent B. Separation of the nucleoside mixture was performed using a flow rate of 0.35 mL/min and the gradient viewed in **Table S1**.

**Table S1**. Utilized HPLC gradient for separation of nucleosides

| **Time [min]** | **A [%]** | **B[%]** |
| --- | --- | --- |
| 0 | 100 | 0 |
| 10 | 92 | 8 |
| 20 | 60 | 40 |
| 23 | 100 | 0 |
| 30 | 100 | 0 |

The main nucleosides (C, U, G, A) were analyzed photometrically at 254 nm by the DAD module whereas Tm was detected *via* the mass spectrometer operating in the positive ion mode. The corresponding ESI settings are depicted in **Table S2**.

**Table S2**. ESI settings

| **Parameter** |  |
| --- | --- |
| Gas temperature | 350 °C |
| Gas flow | 8 L/min |
| Nebulizer pressure | 50 psi |
| Sheath gas temperature | 350 °C |
| Sheath gas flow | 12 L/min |
| Capillary voltage | 3000 V |

Agilent MassHunter Qualitative Analysis software (Version B.05.00) was used in the DMRM (dynamic multiple reaction monitoring) mode for the quantification of the Tm content. Further information can be seen in **Table S3**.

**Table S3**. QQQ settings for Tm analysis

| **Molecular weight [Da]** | **Precursor ion [m/z]** | **Product ion [m/z]** | **Fragmentor Voltage [V]** | **Collision Energy [eV]** | **Cell accelerator voltage [V]** | **Retention Time [min]** |
| --- | --- | --- | --- | --- | --- | --- |
| 272 | 273 | 127 | 66 | 5 | 2 | 14.3 |

The detailed procedure for the Tm quantification was as follows:

First, the peak areas (AUC) from the recorded UV chromatograms (sample and calibration) of the main nucleoside guanosine were extracted. Here, guanosine dilutions (0.5 – 500 pmol) were utilized to obtain a calibration curve which enabled the calculation of a guanosine calibration factor (slope of the linear regression equation). By using this factor, the injected amount of guanosine (in pmol) in the samples could be achieved. To obtain the exact quantity of injected RNA, the calculated guanosine amount was divided by the number of guanosines (here: 23) in the corresponding RNA sequence.

The modified nucleoside was quantified by integrating the corresponding MS/MS peaks (sample and calibration). Here, calibration dilutions of Tm ranging from 10 to 1000 fmol were measured to receive the Tm calibration factor which enabled the determination of modification amount in fmol. In the last step, this quantity was correlated with the injected RNA amount to obtain the Tm/RNA ratio. Standard deviations were achieved by measuring technical replicates.


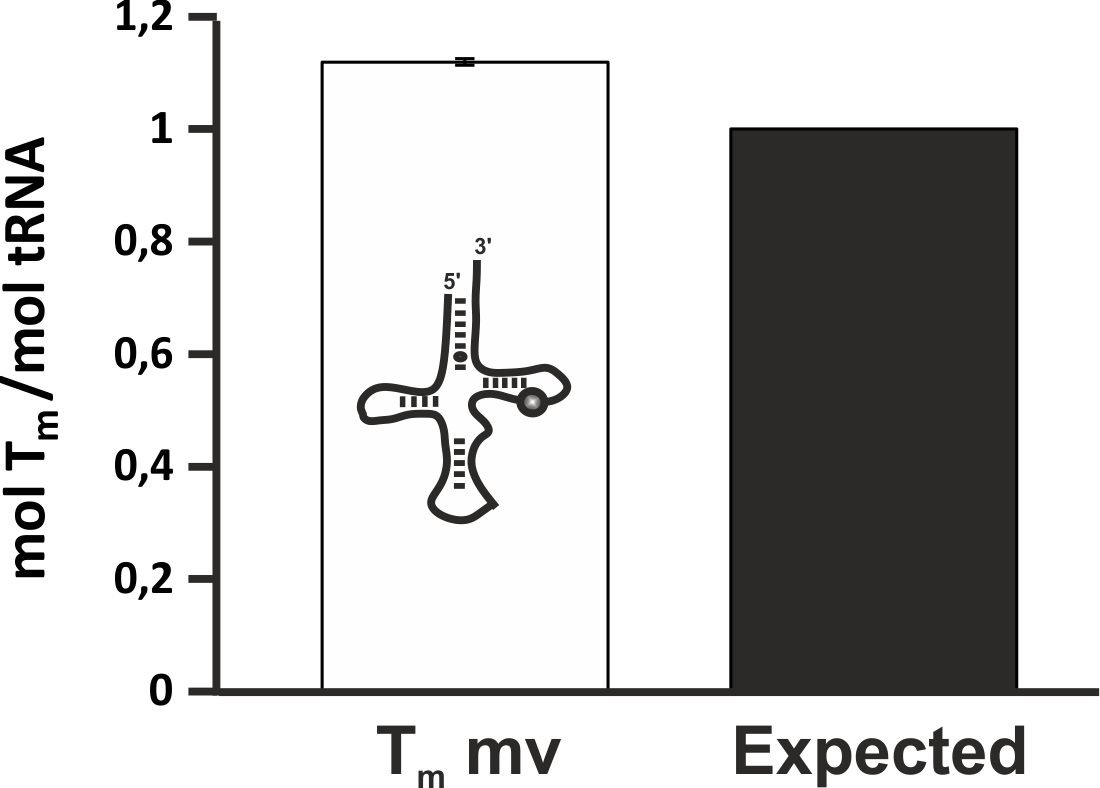


**Figure S2. LC-MS/MS analysis of Tm mv.** Examination of the created Tm modivariant using a QQQ mass spectrometer revealed the presence of one Tm molecule per molecule RNA indicating the suitability of this tRNA for immunostimulation tests.

**RiboMethSeq analysis of X(m) modivariants**

Position and relative stoichiometry of 2’-O-Me nucleotides in modivariants were assessed by previously described tRNA-RiboMethSeq (2). In brief, 100 ng of synthetic modivariant tRNA were fragmented in 50 mM bicarbonate buffer pH 9.2 for 12 min at 96°C, the reaction was stopped by NaOAc pH 4.2, at a final concentration of 0.3 M followed by ethanol precipitation. Alkaline fragments were end-repaired and converted to sequencing library by NEBNext Small RNA Library Prep Set for Illumina (NEB, USA). Sequencing was performed in single-read SR50 nt mode, ~5-8 mln of reads were collected for each library. Further treatment was performed as described for *E. coli* and yeast tRNA analysis (2), the sequence of human tRNA^Lys^_3_ (or respective point mutant) was used as a reference for sequence alignment. Calculations of RiboMethSeq scores were performed using 5’- and 3’-end reads’ information and taking into account values for 2 neighboring positions to the modified residue. The graph shows the Methylation Score (MethScore) values representing relative stoichiometry of modification at position 54. No other hits corresponding to potential 2’-O-Me residues were detected by this analysis.


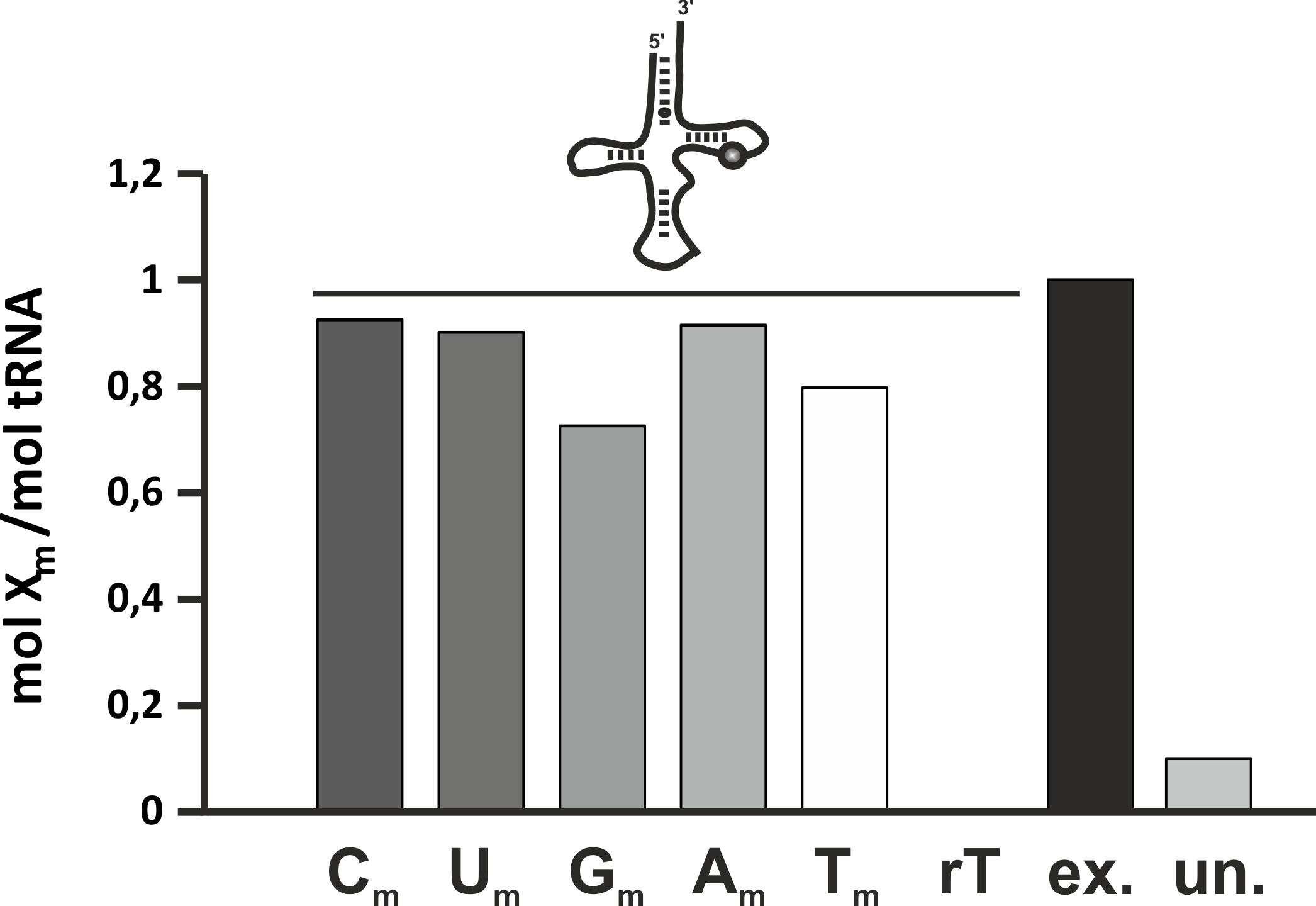


**Figure S3. RiboMethSeq analysis of X(m) mv.** Amount of incorporated modifications in tRNA^Lys^_3_ modivariants was measured *via* RiboMethSeq. Abbreviations: 2’-O-methylcytidine (Cm), 2’-O-methyluridine (Um), 2’-O-methylguanosine (Gm), 2’-O-methyladenosine (Am), 2’-O-methylthymidine (Tm), Ribothymidine (Tm), expected (ex.), unmodified (un.).

**REFERENCES**

1. Crain, P.F. (1990) Preparation and enzymatic hydrolysis of DNA and RNA for mass spectrometry. *Methods Enzymol*, **193**, 782-790.

2. Marchand, V., Pichot, F., Thuring, K., Ayadi, L., Freund, I., Dalpke, A., Helm, M. and Motorin, Y. (2017) Next-Generation Sequencing-Based RiboMethSeq Protocol for Analysis of tRNA 2'-O-Methylation. *Biomolecules*, **7**.
